# Coronary flow reserve correlates with right ventricular dysfunction and predicts right heart failure in patients with pulmonary arterial hypertension

**DOI:** 10.1186/1532-429X-13-S1-P329

**Published:** 2011-02-02

**Authors:** Jan Skrok, Monda L Shehata, Stephen Mathai, Miguel Santaularia Tomas, Sukhminder Singh, Reda E Girgis, James O Mudd, Danielle Boyce, Noah Lechtzin, Joao AC Lima, David A Bluemke, Paul M Hassoun, Jens Vogel-Claussen

**Affiliations:** 1 Johns Hopkins University School of Medicine, Baltimore, MD, USA

## Background

In pulmonary arterial hypertension (PAH) increased pressure and resistance in the pulmonary vascular bed cause right ventricular (RV) hypertrophy with increased myocardial oxygen demand. Studies in animals with RV hypertrophy demonstrated maintained resting but reduced stress perfusion and coronary perfusion reserve, which may contribute to RV failure.

## Purpose

The purpose of our study was to investigate myocardial perfusion by comparing coronary sinus flow reserve (CFR) between PAH patients and healthy controls and to correlate CFR with biventricular function and pulmonary hemodynamics using magnetic resonance imaging (MRI).

## Methods

**Figure 1:**
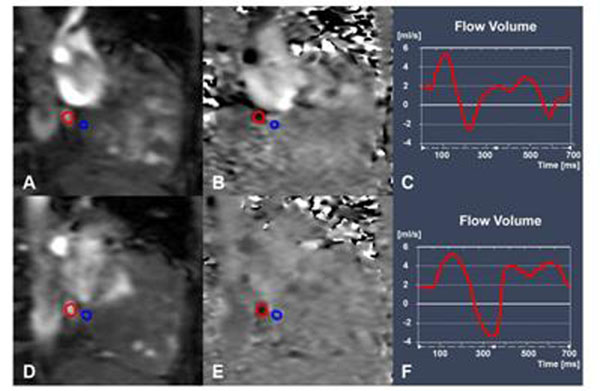
ROI Placement and Flow-Time-Curves. Phase contract MRI images of the CSF for a patient with scleroderma-associated PAH (mPAP 49 mmHg) during rest (top row: A, B) and adenosine-induced stress (bottom row: D, E). The red ROI is drawn around the coronary sinus, the blue ROI is placed in adjacent myocardium to correct for through-plane motion. The flow-time curves (C, F) demonstrate that net CSF increased only slightly from rest (0.86 ml/min/g) to stress (1.32 ml/min/g), resulting in a CFR of 1.53. Correspondingly, there is only little change in the diameter of and flow signal within the coronary sinus.

Thirty-one patients with known or clinically suspected PAH underwent right heart catheterization (RHC) and 3T cardiac MRI on the same day. Twenty patients were found to have PAH, eleven patients did not have PAH. Seventeen age- and gender-matched healthy volunteers were also studied with MRI. Rest and adenosine-stress coronary sinus flow (CSF) were measured with phase contrast MRI (Figure 1) and adjusted to the biventricular mass. Resting CSF was normalized for the rate-pressure-product (RPP = systolic blood pressure x heart rate / 10,000; CSF_norm_ = CSF/RPP). CFR was calculated by dividing stress CSF by resting CSF_norm_ (CFR = Stress CSF / Rest CSF_norm_).

## Results

**Figure 2:**
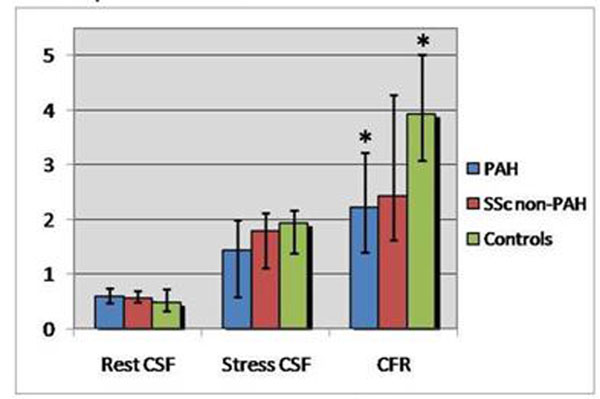
Resting and Stress Coronary Sinus Flow and Coronary Flow Reserve. Bar graphs demonstrate median values and 25^th^-75^th^ percentiles (error bars) for resting CSF (left), stress CSF (middle) and CFR (right) for each group. Values for PAH patients are shown in blue, those for SSc non-PAH patients in red and those for the control group in green. PAH patients demonstrated a trend toward higher resting and lower stress CSF as well as a significantly lower CFR (*p=0.008) compared to healthy controls. The Non-PAH group demonstrated values in-between those for PAH patients and controls.

CFR for PAH patients was significantly lower (2.22 [1.40-3.22]) than for controls (3.93 [3.07-5.01], p=0.002) (Table 1, Figure 2) and was inversely correlated with mean pulmonary arterial pressure (r=-0.48, p=0.03), pulmonary vascular resistance index (r=-0.47, p=0.04), RV end-diastolic and end-systolic volume/BSA (r=-0.68, p=0.0009 and r=-0.60, p=0.005), RV mass/BSA (r=-0.79, p<0.0001), biventricular mass/BSA (r=-0.73, p=0.0002), and ventricular mass index (r=-0.66, p=0.001) (Table 2). On multivariate linear regression analysis, RV mass/BSA was the main predictor of CFR for PAH patients. CFR was able to distinguish between PAH patients with and without right heart failure, defined as RV CI <2.2L/min/m^2^ (area under the ROC curve 0.81 (95% CI: 0.55-1.00); sensitivity 83.3%, specificity of 85.7% for a threshold value of 1.67).

**Table 1:** Resting and stress coronary sinus flow and coronary flow reserve

	PAH	Non-PAH	Controls	p
Rest CSF_norm_ (ml/min/g)	0.59 [0.46-0.74]	0.55 [0.49-0.70]	0.47 [0.33-0.73]	0.42

Stress CSF (ml/min/g)	1.43 [0.58-2.00]	1.80 [1.11-2.11]	1.93 [1.38-2.17]	0.17

CFR	2.22^#^ [1.40-3.22]	2.43 [1.63-4.28]	3.93^#^ [3.07-5.01]	0.008*

**Table 2:** Correlations of CFR with pulmonary hemodynamics as well as biventricular functional and structural parameters

	PAH Patients (n=20)		All Patients (n=31)	
	r	p	r	p

Age	-0.002	1.0	-0.18	0.35

Mean PAP (mmHg)	-0.48	0.03*	-0.39	0.03*

Systolic PAP (mmHg)	-0.28	0.24	-0.33	0.07

PVRI 9Dyne sec/cm^5/m2^)	-0.57	0.04*	-0.37	0.04*

PCWP (mmHg)	-0.39	0.09	-0.36	0.04*

RV Stroke Volume Index (ml/m^2^)	0.39	0.09	0.13	0.50

Cardiac index (l/min//m^>2^)	0.33	0.16	0.17	0.35

RV Stroke Word Index	-0.05	0.82	-0.32	0.08

LV ED Volume Index (ml/m^2^)	0.09	0.71	-0.008	0.97

LV ES Volume Index (ml/m^2^)	0.12	0.62	0.08	0.68

LV Stroke Volume Index (ml/m^2^)	0.05	0.83	-0.09	0.63

LV Cardiac Index (l/min//m^2^)	0.20	0.39	0.22	0.23

LV Ejection Fraction (%)	-0.03	0.89	-0.06	0.74

LV ED Mass Index (g/m^2^)	-0.35	0.13	-0.31	0.08

RV ED Volume Index (ml/m^2^)	-0.68	0.0009*	-0.57	0.0008*

RV ES Volume Index (ml/m^2^)	-0.60	0.005*	-0.55	0.001*

RV Stroke Volume Index (ml/m^2^)	0.02	0.92	-0.09	0.62

RV Cardiac Index (l/min//m^2^)	0.17	0.48	0.18	0.33

RV Ejection Fraction	0.43	0.06	0.36	0.045*

RV ED Mass Index (g/m^2^)	-0.79	<0.0001*	-0.59	0.0004*

Total Biventricular Mass Index (g/m^2^)	-0.73	0.0002*	-0.65	<0.0001*

VMI	-0.66	0.001*	-0.49	0.006*

## Conclusion

PAH patients have a reduced CFR compared to healthy volunteers, which correlates with pulmonary hemodynamics and RV dysfunction. A decreased CFR is predicted by RV mass and may contribute to RV failure. Further studies are warranted to investigate the predictive value of CFR with regards to patient outcome.

